# Histopathological composition of thrombus material in a large cohort of patients with acute ischemic stroke: a study of atypical clots

**DOI:** 10.3389/fneur.2025.1563371

**Published:** 2025-03-20

**Authors:** Laura Rojas-Bartolomé, María Payá, Rosa Barbella-Aponte, Laura Restrepo Carvajal, Jorge García-García, Oscar Ayo-Martín, Juan David Molina-Nuevo, Gemma Serrano-Heras, Enrique Juliá-Molla, María José Pedrosa-Jiménez, Lorena López-Martínez, Ángela Fernández López, Tomás Segura, Francisco Hernández-Fernández

**Affiliations:** ^1^Department of Neurology, University General Hospital of Albacete, Albacete, Spain; ^2^Department of Surgical Pathology, University General Hospital of Albacete, Albacete, Spain; ^3^Department of Radiology, University General Hospital of Albacete, Albacete, Spain; ^4^Research Unit, University General Hospital of Albacete, Albacete, Spain; ^5^Medical School, Biomedicine Institute (IB), University of Castilla-La Mancha (UCLM), Albacete, Spain

**Keywords:** atypical clot, ischemic stroke, mechanical thrombectomy, histopathological, septic emboli

## Abstract

**Introduction:**

Clot composition appears to be associated with outcomes in stroke recanalization therapy. This study aims to analyze thrombus composition and assess the relationship between atypical clot composition and clinical parameters, laboratory markers, and recanalization strategies in a series of patients with acute ischemic stroke (AIS) undergoing endovascular treatment (EVT).

**Methods:**

This is a prospective single-center registry conducted from December 2014 to July 2022. All retrieved clots were examined under an established protocol and classified as follows: red blood cell-rich clots (RBC), fibrin/platelet-rich clots (FPC), mixed clots (MC), septic emboli (SE), atheroma emboli (AE), fatty emboli (FE), and calcium emboli (CE). We categorized them into two groups: atypical clot composition (ACC: SE, AE, FE, and CE) and usual clot composition (UCC: RBC, FPC, and MC). A subgroup of 10 ACC (four SE, three AE, two FE, and one CE) and nine UCC (three RBC, three FPC, and three MC), matched by age and sex, was analyzed using immunohistochemistry to detect neutrophil extracellular traps (NETs).

**Results:**

A total of 606 patients were assessed for EVT, with 448 (73.92%) meeting the inclusion criteria. The clot categorization was as follows: FPC 211 (47.1%), RBC 105 (23.4%), MC 104 (23.2%), SE 16 (3.6%), AE 5 (1.1%), CE 4 (0.9%), and FE 3 (0.7%). Consequently, we classified 420 (93.75%) patients into the UCC group and 28 (6.25%) into the ACC group. Bivariate analysis revealed that the ACC group had a significantly higher number of leukocytes (11.40 leukocytes/mm^3^ vs. 9.49, *p* = 0.005), a greater frequency of TICA occlusion (28.6% vs. 9.8%, *p* = 0.006), and higher mortality at three months (28.6% vs. 12.4%, *p* = 0.038). Multivariate analysis indicated that atypical clot composition was significantly associated with a higher prevalence of diabetes mellitus, smoking, occlusion of the terminal internal carotid artery, and an increased number of passes. Immunohistochemical studies showed the presence of neutrophil extracellular traps (NETs) in all 19 thrombi that were analyzed.

**Conclusion:**

Diabetes and TICA occlusion were the strongest predictors of atypical clot composition. We also observed a significant association between atypical composition and an increased number of passes. Furthermore, the presence of NETs in all thrombi analyzed, regardless of their composition, indicates inflammatory mechanisms associated with clot formation and consolidation in AIS.

## Introduction

There is a growing body of evidence showing that stroke thrombi are complex and heterogeneous. Numerous studies correlate clot composition with functional parameters, neuroimaging, laboratory markers, and the recanalization technique (1–10).

Factors such as the mechanical properties of clots and their histological composition are relevant to successful reperfusion (11). Red blood cell-rich clots (RBC) are easier to remove than fibrin/platelet-rich clots (FPC), likely due to their lower coefficient of friction (12, 13).

Further studies have revealed greater complexity regarding the types and compositions of clots, extending beyond platelets, erythrocytes, and fibrin. Additionally, a small proportion of patients present with atypical clot compositions. Septic emboli (SE) have been associated with increased clot stiffness. On the one hand, bacteria can modify the microstructure of fibrin. In addition, the infectious process facilitates the influx of neutrophils that release DNA and histones through the phenomenon of NETosis. NETs have been clearly associated with clots that have a tighter microstructure, which is resistant to thrombolysis and thrombectomy treatments (14–16). The prognosis for stroke caused by SE, such as infective endocarditis (IE), is typically unfavorable (17, 18). Moreover, calcic emboli (CE) are stiffer than clots composed mainly of fibrin and erythrocytes, which may influence recanalization (19). Additionally, they are associated with worse angiographic outcomes and higher mortality rates compared to patients with non-calcified thrombi (20–22).

Endovascular treatment (EVT) has become the standard care for patients with acute ischemic stroke (AIS) and large vessel occlusion (LVO) (23). However, no robust evidence is available to demonstrate the benefit of mechanical thrombectomy (MT) in SE-related strokes (24), as clinical evidence is limited to a few case reports published to date (25–32). The use of MT in CE-related strokes in several case series showed low recanalization rates, but there are no recommendations to improve the procedure and final outcome (20, 22).

Despite the different series published about clot composition, we currently do not have consistent data on the incidence of atypical thrombus. A previous study of our group showed a 6.2% incidence of SE originating from both IE and other infectious foci (33); the CE incidence has been estimated between 1.3 and 5.9% (20, 34). To our knowledge, there are no published data on the incidence of FE and AE.

In this study, using an extensive unicentric series of MT, we aim to investigate the frequency of atypical composition thrombi and the potential influence that histological architecture differences could have on the process of thrombus mechanical removal and patient functional prognosis.

Furthermore, given that neutrophils and NETs are essential in the early response to pathogens and acute inflammation (33), as previously reported (34, 35) in sterile thrombus formation, we considered it of interest to study the presence of NETs in our series.

## Materials and methods

### Study design and patient selection

We conducted a retrospective, observational, unicentric analysis of a prospective database comprising AIS patients who underwent EVT at a tertiary stroke center from December 2014 to July 2022. All patients included followed our institutional protocols, which are based on current international stroke guidelines.

This study was approved by the local Clinical Research Ethics Committee, with reference number (2019/03/031). All patients included in the study or their relatives provided written informed consent, allowing for the entry of their information into our reperfusion registry and the subsequent use of the data for scientific purposes, in accordance with Spanish Personal Data Protection law.

For our study, patients were selected in case they met the following criteria: (1) substantial neurological deficit (generally NIHSS≥6) and ischemic stroke with confirmed large or medium vessel occlusion in cerebral angiography; (2) time from symptom onset to groin puncture <24 h, including wake-up strokes and those of uncertain onset; (3) no intracranial hemorrhage on baseline cranial tomography (CT); (4) for patients with time from symptom onset to EVT > 6 h, presence of target mismatch profile on CT perfusion; and (5) availability of thrombus removal for histological analysis. If no contraindication existed, previous treatment with intravenous recombinant tissue-type plasminogen activator (IV r-tPA) was administered prior to EVT. The inclusion criteria for endovascular treatment have not changed significantly since their establishment in 2014 (33).

**Figure 1:**
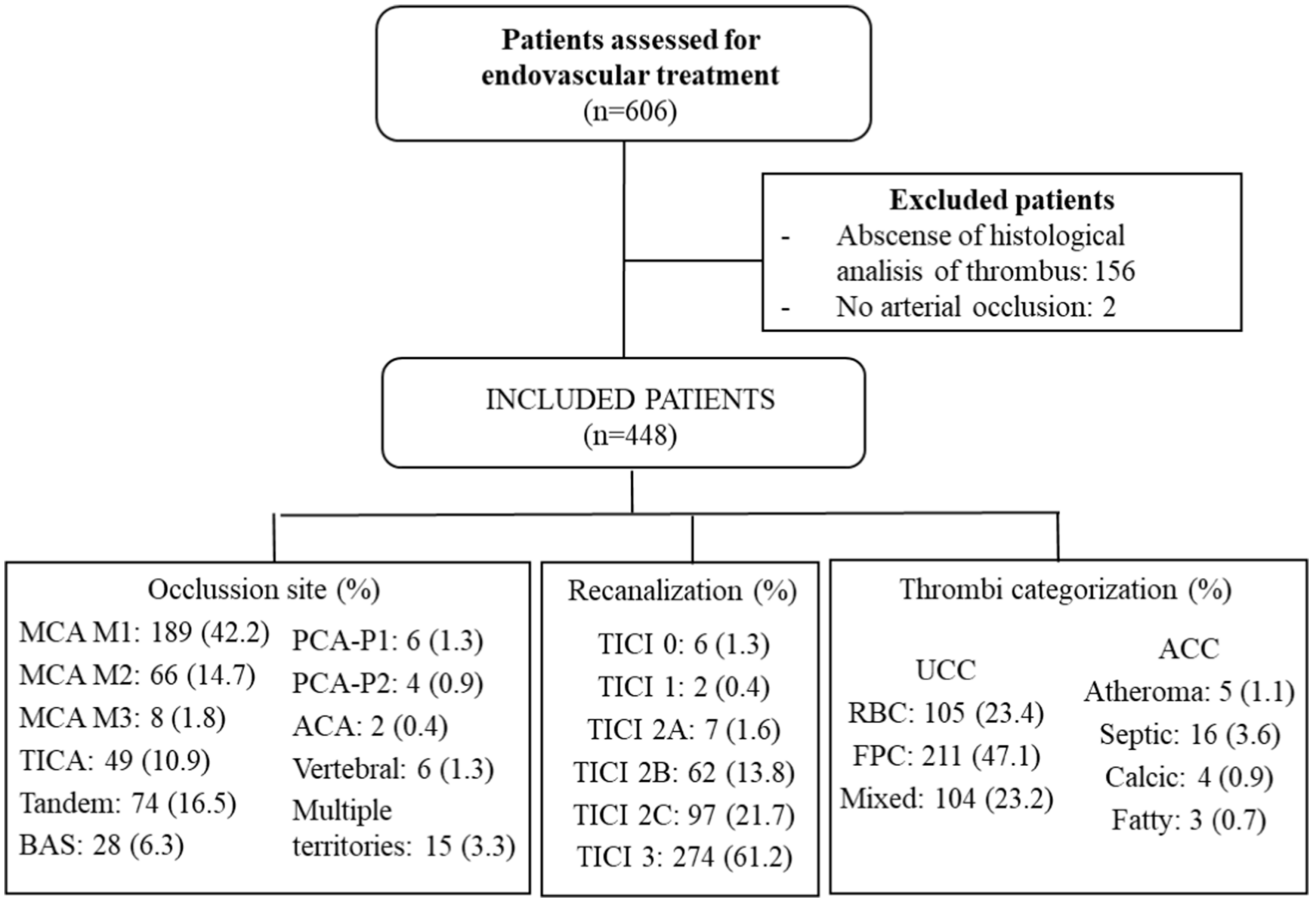
A flowchart with the results of included patients. MCA, middle cerebral artery; TICA, terminal internal carotid artery; BAS, basilar; PCA-P1, posterior cerebral artery P1 segment; PCA-P2, posterior cerebral artery P2 segment; ACA, anterior cerebral artery; TICI, thrombolysis in cerebral infarction; UCC, usual clot composition; ACC, atypical clot composition; RBC, red blood clot; and FPC, fibrin-predominant clot.

All retrieved clots were analyzed according to a standard protocol that included histopathological and bacteriological studies. Figure 1 illustrates the flowchart for patient inclusion.

### Clinical data and variables collected

The following baseline variables were collected from our reperfusion registry: demographics (age, sex), vascular risk factors, and comorbidities (high blood pressure, diabetes mellitus, hypercholesterolemia, ischemic heart disease, active smoking, atrial fibrillation, intermittent claudication), stroke etiology based on the TOAST classification (35), arterial occlusion point, presence of the hyperdense middle cerebral artery sign according to the baseline CT, blood laboratory markers (C-reactive protein-CRP-and leukocyte count), clinical evaluation using the NIHSS (National Institutes of Health Stroke Scale) on admission and at discharge (relative to the baseline score), the baseline modified Rankin Scale (mRS), neurological outcome rates at 3 months [considering a favorable outcome to be mRS ≤ 2 and mortality mRS = 6), use of intravenous (IV) thrombolysis, time from onset to groin puncture, procedural EVT variables (time from groin puncture to completion of the procedure, type of endovascular device, number of passes, placement of intracranial definitive stents, and recanalization according to the TICI (Thrombolysis in Cerebral Infarction) scale], with a successful recanalization rate defined as TICI≥2B and procedural safety (symptomatic intracranial hemorrhage-sICH-and mortality). We have defined refractory thrombectomy (RT) as requiring three or more passes (36).

Regarding SE, variables related to the following were collected: microbiological profile, endocarditis complications (including cardiac failure, renal failure, septic shock, and cardiac surgery), and concomitant infections other than endocarditis (such as pneumonia, urinary tract infection, and sepsis of unknown origin).

### Neuroimaging protocol

A non-contrast basal cranial CT was performed before EVT. Data on early ischemia were evaluated on the basal CT using ASPECTS (Alberta Stroke Program Early CT Score). Arterial occlusion was assessed using an angio-CT scan, and mismatch was determined by CT perfusion. All CT imaging was conducted with a 64-slice Philips Brilliance CT (Koninklijke Philips Electronics N.V., Amsterdam, Netherlands), and the images were analyzed by a neuroradiologist. A control CT was performed 24 h after the procedure or in the event of neurological deterioration to assess for the presence of an established infarct or hemorrhagic transformation.

Symptomatic intracranial hemorrhage (sICH) is defined by the Heidelberg criteria (37) as any intraparenchymal hemorrhage associated with an increase of 4 or more points on the NIHSS scale or resulting in death.

### Endovascular procedures

Endovascular procedures were performed using either an Azurion 7 B20/15 biplane model (Koninklijke Philips Electronics N.V., Amsterdam, Netherlands) or a monoplane angiography Innova GE model (General Electric Company, Schenectady, NY, United States), conducted by a board-certified neuroradiologist. General anesthesia was administered systematically. Vascular access was typically achieved by puncturing the right femoral artery with the Seldinger technique. A Flowgate 8F (Stryker Corporation, Kalamazoo, MI, United States) guide catheter was used in the cervical segment of the ICA during anterior circulation stroke. Following this maneuver, hyperselective catheterization of the occluded vessel was performed, and the clot was crossed with a Traxcess 0.014′′ microguide (MicroVention Inc., Tustin, CA, United States) and a Trevo Pro (Stryker Corporation, Kalamazoo, MI, United States) 0.021′′ microcatheter. For M1, M2, and intracranial ICA occlusions, the selected stent retrievers (SR) included Solitaire FR (Medtronic, Dublin, Ireland), Trevo XP (Stryker Corporation, Kalamazoo, MI, United States), or Embotrap II (Johnson & Johnson, New Brunswick, NJ, United States) with dimensions of 4–6 mm. In cases of M2 occlusions immediately anterior to the opercular gyrus or M3 occlusions, a Trevo XP or Catch mini (Balt, Montmorency, France) 3 mm SR was used. The device was removed after 2 min of balloon occlusion, while simultaneous manual aspiration was performed using a 60-cc syringe.

Alternatively, in M1 occlusions, or after SR failure following two passes, an ACE68 or JET7 (Penumbra Inc., Alameda, CA, United States) aspiration catheter was used and connected to an automatic pump for 3 min. The device was then removed through simultaneous manual aspiration using a 60-cc syringe. If the results were unsatisfactory after two aspirations, a combined approach involving SR and local aspiration was employed. After each pass, the devices, along with the intermediate catheter, were withdrawn, and the aspiration syringe was inspected for thrombus fragments. The device was gently rinsed with heparinized saline to eliminate any thrombus fragments. The aspirated material was gently flushed with saline to detect any smaller fragments. For posterior circulation occlusions, aspirate thrombectomy was chosen as the primary therapeutic option.

Once the thrombectomy is concluded, control angiographic series in anteroposterior and lateral projections are performed, and the TICI scale is determined.

### Histopathological, bacteriological, and immunohistochemical study

The clots were stored in 4% formaldehyde and sent to the Pathology Department. They were embedded in paraffin and sectioned into 4-μm slices using a microtome. Consecutive sections were processed according to established histological and histochemical protocols, which included Hematoxylin–Eosin, periodic acid–Schiff (PAS), Gram, and Gomori’s Trichrome stains.

An expert neuropathologist conducted this study by analyzing the macroscopic and microscopic characteristics of the clots at 2× (scale bar 500 μm), 4× (scale bar 250 μm), 10× (scale bar 100 μm), and 20× (scale bar 50 μm) magnification. The pathologist then examined thrombus sections stained with hematoxylin and eosin (H&E) under a light microscope to evaluate their composition, focusing on the following: red blood cells, white blood cells (WBCs), including the number of polymorphonuclear cells and their status, fibrin/platelet distribution, the degree of thrombus organization, and other components such as calcium, cholesterol crystals, fat, and the presence of bacteria. A semi-quantitative assessment was performed, as previously described by our group (2), through visual analysis using a method where an imaginary grid was overlaid on the slides to systematically analyze different regions of the thrombus. The clots were categorized into seven types based on the following criteria.

- Red blood clot (RBC): it occurs when the percentage of red blood cells is ≥60%. A fibrin-predominant clot (FPC) is identified when the ratio of fibrin to platelet is ≥60%. A mixed clot (MC) is present if there is no clear predominance of these components.
- Septic emboli (SE): they occur when there is an increased number of white blood cells (WBCs) exhibiting morphological alterations. To detect bacteria, the slide was examined at 100× magnification (scale bar, 10 μm) using immersion oil.
- Atheroma emboli (AE): they consist of precipitated cholesterol crystals attached to fragments of blood vessels. They are accompanied by a macrophage-mediated inflammatory response.
- Calcic emboli (CE): they are a major component of calcified tissue.
- Fatty emboli (FE): they are an accumulation of adipose tissue as the major component.

Overall, the clots were divided into two main groups based on their composition and frequency of occurrence: atypical clot composition (ACC), which includes SE, AE, CE, and FE, due to lower frequency, and usual clot composition (UCC), consisting of RBC, FPC, and MC.

**Figure 2:**
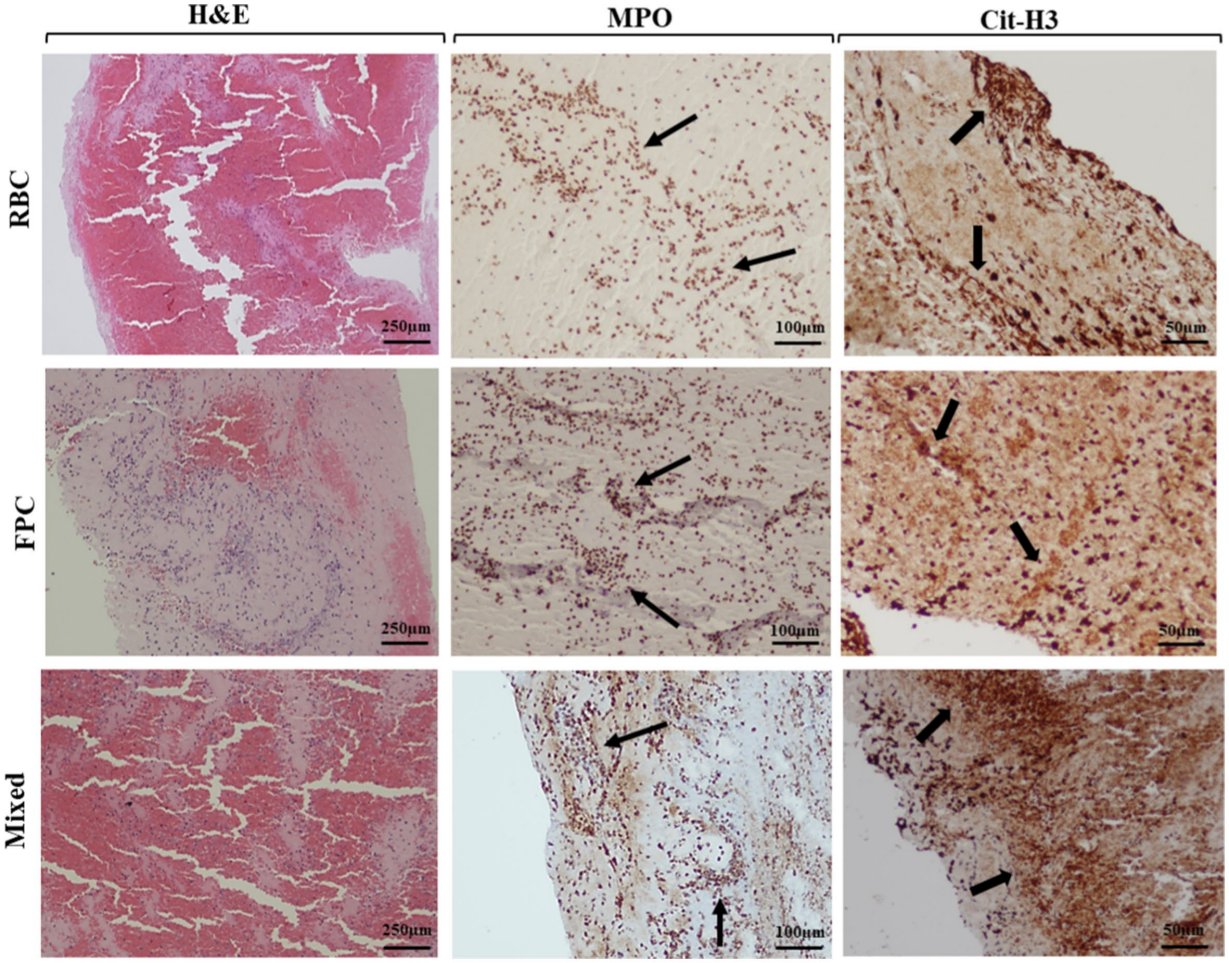
Histological and immunohistochemical study of usual thrombi. Hematoxylin–Eosin (H&E) stain (4× magnification, scale bar, 250 μm). Red blood clot (RBC): red blood cells ≥60%. Fibrin-predominant clot (FPC): fibrin/platelet ≥60%. Mixed clot: no clear predominance of components. Neutrophil extracellular trap (NET) components (indicated by arrows), myeloperoxidase (MPO), and citrullinated histone H3 (Cit-H3) were detected by immunohistochemistry at 10× (scale bar: 100 μm) and 20× (scale bar: 50 μm) magnification, respectively.

**Figure 3:**
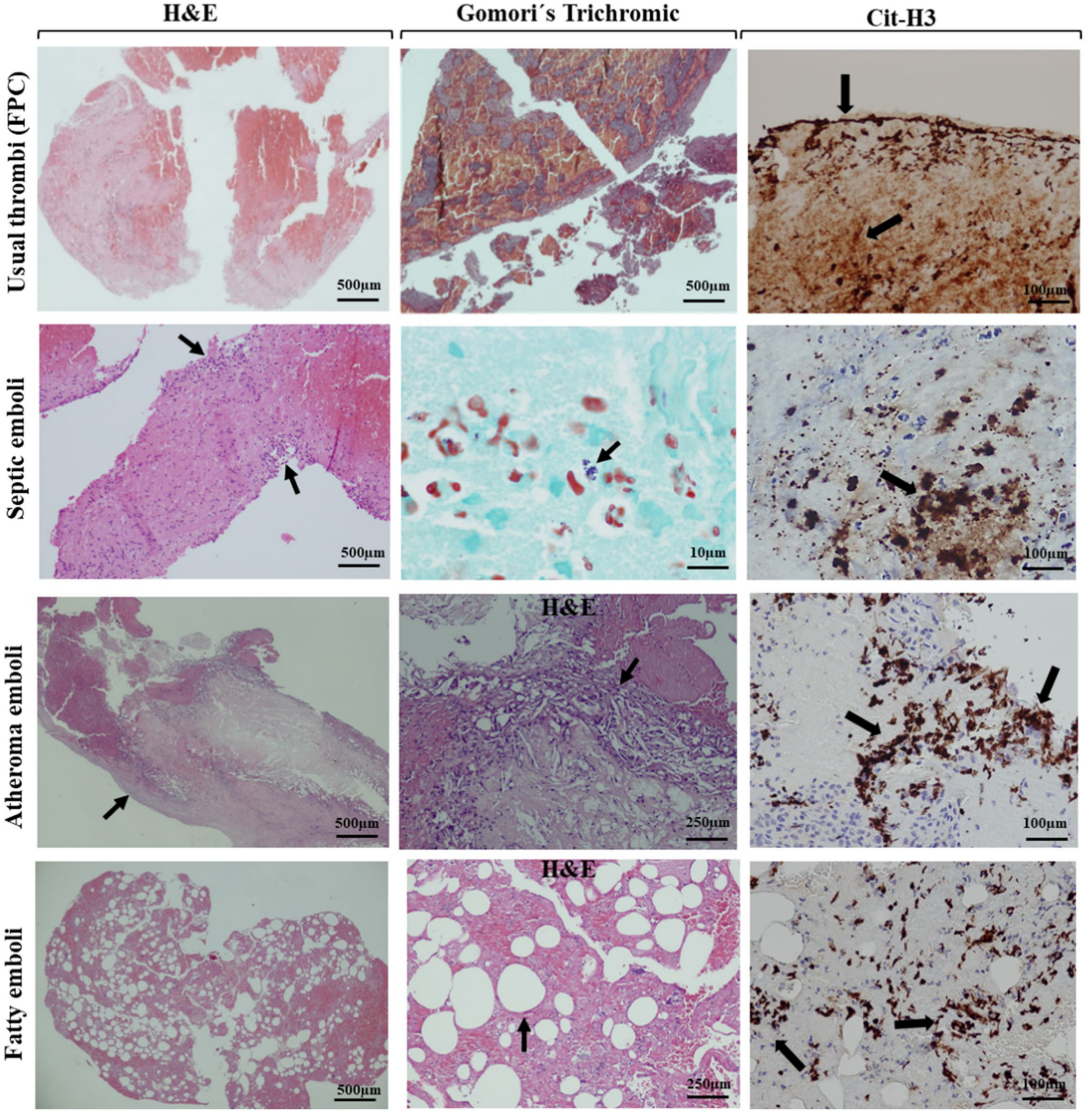
Histological features and NETs detection of a usual clot and atypical thrombi. H&E stain at 2× magnification (scale bar: 500 μm) and 4× magnification (scale bar: 250 μm) of a usual fibrin-rich clot, septic emboli, a fibrin-platelet-predominant clot with accumulated necrotic cells at the periphery (arrows), atheroma emboli with the blood vessel wall (arrow), and precipitated cholesterol crystals (arrow), and fatty emboli with fat cells (arrow). Gomori’s trichrome stain at 100× with immersion oil was used to analyze bacterial cocci accumulation (arrow). Representative images of Cit-H3 staining of different clots (20× magnification, scale bar, 50 μm), highlighting the accumulation of extracellular traps released by neutrophils (arrows).

In 19 thrombi, immunohistochemistry for myeloperoxidase (MPO) and citrullinated histone H3 (Cit-H3) stains were performed as biomarkers of NET formation (38). Specifically, we analyzed 10 atypical thrombi (4 SE, 3 AE, 2 FE, and 1 CE) from those available in the sample based on their prevalence in the large cohort. The remaining nine typical thrombi (3 RBC, 3 FPC, and 3 MC) were paired with the atypical ones from the available samples following the clinical protocol, matching by age and sex; however, there was a slightly higher proportion of women in this group. As shown, Figure 2 presents representative images of typical thrombi, including RBC, FPC, and mixed thrombi, stained with H&E, alongside immunohistochemical analysis using MPO and Cit-H3. In contrast, Figure 3 compares a typical clot (UCC) with representative images of septic, atheromatous emboli, and fatty emboli, also stained with H&E and analyzed using anti-Cit-H3 antibodies. These images highlight the characteristics of these thrombus types.

The protocol for the immunohistochemical detection of myeloperoxidase (MPO) and citrullinated histone H3 is described as follows. Briefly, tissue specimens were collected and fixed in 10% neutral-buffered formalin for 24 h at room temperature, followed by dehydration through a graded ethanol series, clearing in xylene, and paraffin embedding. Sections of 4 μm thickness were cut using a microtome and mounted on positively charged glass slides. For immunohistochemical staining, the slides were processed using the Dako Omnis automated stainer (Agilent Technologies), including deparaffinization and rehydration steps. Heat-induced epitope retrieval (HIER) was performed with EnVision™ FLEX Target Retrieval Solution (pH 6.0) for citrullinated histone H3 and pH 8 for MPO at 97°C for 20 min. Endogenous peroxidase activity was blocked using EnVision™ FLEX Peroxidase-Blocking Reagent for 10 min. The sections were then incubated with either a pre-diluted polyclonal rabbit anti-citrullinated histone H3 (positions R2 + R8 + R17) antibody (ANACROM Diagnóstico, #AP100108) or a ready-to-use polyclonal rabbit anti-MPO antibody. Detection was performed using the EnVision™ FLEX+ Detection System (Agilent Technologies) according to the manufacturer’s protocol. The slides were counterstained with hematoxylin for 4 min, followed by dehydration through graded alcohols, clearing in xylene, and mounting with synthetic resin. Positive control tissues expressing citrullinated histone H3 were included, and negative controls were processed without the primary antibody to ensure staining specificity.

### Statistical analysis

Continuous data were expressed using descriptive statistics (mean, standard deviation, median, and interquartile range). Categorical variables were expressed using percentages.

All quantitative variables exhibited non-normal distribution in the Kolmogorov–Smirnov test. The comparison of quantitative variables was conducted using the Mann–Whitney *U* test. The comparison of categorical variables between two or more subgroups was performed using Pearson’s chi-squared test (*X*^2^) or, where applicable, the Fisher exact test.

The variables included in the bivariate analysis were sex, age, TOAST etiology, high blood pressure (HBP), DM, hypercholesterolemia, ischemic heart disease, intermittent claudication, active smoking status, oral anticoagulation treatment, atrial fibrillation, hyperdense middle cerebral artery sign, intravenous thrombolysis, arterial occlusion point, TICA occlusion, ASPECTS, basal mRS, 3-month mRS, mortality, refractory thrombectomy, sICH, urgent leukocyte count, urgent CRP count, NIHSS on admission, NIHSS at discharge, time from symptom onset to groin puncture, procedure time of MT, number of passes, TICI, first-pass effect, intracranial stenting, endocarditis complications, and concomitant infections other than endocarditis.

All variables related to the dependent variable (the presence or absence of atypical composition) in the bivariate analysis were included in a multivariate binary logistic regression model. The odds ratios (ORs) and corresponding two-sided 95% confidence intervals (95% CIs) for all risk factors were shown in the multivariate analysis. Model fit and predictive strength were examined using the Hosmer-Lemeshow goodness-of-fit test.

A *p*-value of <0.05 was considered statistically significant for all analyses. All results were analyzed using the statistical software IBM SPSS Statistics Version 22.0 (SPSS, Chicago, IL, United States).

## Results

During the study period, 606 patients were evaluated for endovascular treatment (Figure 1), of which 158 were excluded: 156 due to the inability to obtain sufficient thrombus material for analysis and 2 for not having an acute stroke. As a result, 448 patients (73.92%) were ultimately included in our study.

The mean overall age of the sample was 69.6 years (SD 12.87), with 44.4% of patients being women. According to the stroke etiology classification (TOAST), 237 patients (52.9%) were classified as cardioembolic, 117 (26.1%) as indeterminate, 76 (17%) as atherothrombotic, and 18 (4%) due to uncommon etiology. Table 1 presents the distribution of baseline variables, including demographics, vascular risk factors, clinical data, laboratory results, and stroke etiology.

**Table 1:** Distribution of baseline variables (demographics, vascular risk factors, clinical data, laboratory results, and stroke etiology) in patients with usual (RBC, FPC, and MC) vs. atypical clot composition (septic, calcic, fatty, and atheroma emboli).

Variable		Usual clot composition (*n* = 420)	Atypical clot composition (*n* = 28)	*p*-value
Age, mean (SD)		69.57 (13.01)	69.68 (10.77)	0.720
Sex, female (%)		188 (44.8)	11 (39.3)	0.695
Active smoker (%)		66 (15.7)	11 (39.3)	0.012*
Diabetes Mellitus (%)		105 (25)	14 (50)	0.007*
High blood pressure (%)		278 (66.2)	18 (64.3)	0.839
Hypercholesterolemia (%)		168 (40)	12 (42.9)	0.843
Ischemic heart disease (%)		44 (10.5)	1 (3.6)	0.341
Intermittent claudication (%)		12 (2.9)	1 (3.6)	0.573
Atrial fibrillation (%)		178 (42.4)	8 (28.6)	0.169
TOAST etiology	Cardioembolic	228 (54.3)	9 (32.1)	0.135
	Atheromatous large-vessel disease	69 (16.4)	7 (25)	
	Infrequent	17 (4)	1 (3.6)	
	Undetermined	106 (25.2)	11 (39.3)	
TICA occlusion point		41 (9.8)	8 (28.6)	0.006*
Urgent CRP mg/dL and mean (SD)		46.57 (58.1)	63.55 (70.36)	0.396
Urgent leukocytes count/mm^3,^ mean (SD)		9.49 (3.3)	11.40 (3.8)	0.005*
NIHSS on admission and median (IQR)		18 (12)	16.5 (8.5)	0.659
HMCAS (%)		237 (56.4)	13 (46.4)	0.330

TICA, terminal internal carotid artery; HMCAS, Hyperdense middle cerebral artery sign. *P value indicates statistically significant.

The categorization of thrombi was as follows: FPC 211 (47.1%), RBC 105 (23.4%), MC 104 (23.2%), SE 16 (3.6%), AE 5 (1.1%), CE 4 (0.9%), and FE 3 (0.7%). Consequently, we classified 420 (93.75%) patients in the UCC group and 28 (6.25%) patients in the ACC group. We analyzed the potential association between composition and stroke etiology, but no significant correlation was found.

In the septic thrombi, the histochemical study with gram stain detected structures suggestive of bacteria, which were confirmed in 15 cases (93.75%): infective endocarditis in 4 cases (25%), urinary focus in one case (6.25%), digestive focus in one case (6.25%), respiratory focus in one case (6.25%), and no confirmed focus in nine cases (56.25%). Findings of neutrophil extracellular traps (NETs) formation included immunohistochemical studies (cit-H3 and/or MPO) profile positivity performed in nine UCC (only 2.1% of the total thrombi were analyzed, namely three FPC, three RBC, three MC) and 10 ACC (35.7% of the atypical thrombi were analyzed, specifically four SE, three AE, two FE, and one CE). Figure 2 displays representative images of typical thrombi, including RBC, FPC, and mixed thrombi, stained with H&E, alongside immunohistochemical analysis using MPO and Cit-H3. Figure 3 contrasts a typical clot (UCC) with representative images of septic, atheromatous, and fatty emboli, also stained with H&E and examined using anti-Cit-H3 antibodies to detect NETs. These images highlight the unique characteristics of each thrombus type while demonstrating the common presence of NETs in all of them.

### Bivariate analysis

Table 2 shows the bivariate analysis results. There were no significant differences between the demographic variables, stroke etiology, or clot composition. Additionally, there were no differences in clinical severity parameters (NIHSS) or in-hospital laboratory markers.

**Table 2:** Functional outcome parameters, mortality, and procedural EVT variables.

Variable	Usual clot composition (*n* = 420)	Atypical clot composition (*n* = 28)	*P*-value
Favorable TICI (%)	408 (97.1)	25 (89.3)	0.060
mRS ≤ 2 at 3 months (%)	198 (47.1)	17 (60.7)	0.241
Mortality (mRS = 6 at 3 months) (%)	52 (12.4)	8 (28.6)	0.038*
sICH (%)	15 (3.6)	2 (7.1)	0.288
Number of passes, median (IQR)	1 (2)	2 (3.75)	0.018*
Procedure time, mean min (SD)	40.9 (53.11)	59.68 (50.51)	0.057
Refractory Thrombectomy	110 (26.2)	12 (42.9)	0.077

Bivariate analysis. Favorable TICI (thrombolysis in Cerebral Infarction): ≥2B. mRS (modified Rankin Scale at baseline). sICH, symptomatic intracranial hemorrhage. *P value indicates statistically significant.

We observed a higher prevalence of DM in the ACC group (50% ACC vs. 25% UCC, *p* = 0.004) and active smokers (39.3% ACC vs. 15.7% UCC, *p* = 0.012) but no significant association with the rest of the vascular risk factors or comorbidities. ACC also showed an increased number of leukocytes (SD) [11.40 leucocytes/mm^3^ (3.89) ACC vs. 9.49 (3.36) UCC, *p* = 0.005].

Regarding procedural EVT variables, functional outcome parameters, and mortality, the TICA occlusion point was more common in the ACC group (28.6% ACC vs. 9.8% UCC, *p* = 0.006). ACC was significantly associated with a higher number of passes at MT (2 ACC vs. 1 UCC, *p* = 0.018). Refractory thrombectomy (three or more passes) occurred more frequently in the ACC group, with 42.9% in ACC compared to 26.2% in UCC (*p* = 0.07). The first-pass effect was less frequent in ACC than in UCC (35.7% in ACC vs. 54% in UCC, *p* = 0.07), while the procedure time was longer (40.90 min vs. 59.68 min, *p* = 0.057); however, these differences were not statistically significant. Additionally, ACC was significantly associated with higher mortality at 3 months (28.6% in ACC vs. 12.4% in UCC, *p* = 0.038).

In the SE group, we compared the four cases (25%) of IE with the remaining infections (one urinary, one digestive, and one respiratory focus) and nine cases (56.25%) with no confirmed infection origin. There were no differences in demographic variables, vascular risk factors, comorbidity, or stroke etiology. We also found no differences in recanalization strategies or functional outcomes based on the infection’s origin.

### Multivariate analysis

In the multivariate analysis (Table 3), the presence of DM (50% ACC vs. 25% UCC, OR = 2.780; 95% CI 1.203–6.427; *p* = 0.017), active smoking (39.3% ACC vs. 15.7% UCC, OR = 1.708; 95% CI 1.014–2.878; *p* = 0.044), and TICA occlusion (28.6% ACC vs. 9.8% UCC, OR = 3.911; 95% CI 1.490–10.268; *p* = 0.006) were significantly associated with atypical clot composition.

**Table 3:** Variables related to atypical composition in our series.

Variable	Usual thrombus composition (*n* = 420)	Atypical thrombus composition (*n* = 28)	Exp (B)	OR (IC 95%)	*P*-value
DM (%)	105 (25)	14 (50)	2.780	1.203–6.427	0.017*
Active smoker (%)	66 (15.7)	11 (39.3)	1.708	1.014–2.878	0.044*
TICA occlusion (%)	41 (9.8)	8 (28.6)	3.911	1.490–10.268	0.006*
Number of passes, median (IQR)	1 (2)	2 (3.75)	1.230	1.002–1.511	0.048*
Mortality (mRS = 6 at 3 months) (%)	52 (12.4)	8 (28.6)	1.892	0.709–5.050	0.203
Urgent Leukocytes count/mm^3,^ mean (SD)	9.49 (3.3)	11.40 (3.8)	1	1–1	0.065

Multivariate analysis. DM, diabetes mellitus; and TICA, terminal internal carotid artery. *P value indicates statistically significant.

The ACC group had a significantly higher number of passes [median (IQR) 2 (3.75) ACC vs. 1 (2) UCC, OR = 1.230; 95% CI 1.002–1.511; *p* = 0.048] compared to the UCC group. We did not find significant differences in mortality or in the other parameters analyzed.

## Discussion

To the best of our knowledge, this study presents the largest collection of histopathological and bacteriological data on thrombi obtained through mechanical thrombectomy (MT), providing insights into the incidence and composition of atypical thrombi, including septic, calcific, atheromatous, and fatty emboli. We identified diabetes mellitus, active smoking, and TICA occlusion as strong predictors of atypical clot composition. Atypical thrombi were associated with a higher number of passes during MT, suggesting that increased procedural NET formation was present in every thrombus analyzed, underscoring the critical role of inflammation in thrombus development during ischemic stroke.

Evidence regarding atypical causes of thrombus composition remains limited. Current knowledge about calcific emboli is primarily based on isolated case reports or small series, where thrombus composition is predominantly estimated through simple cranial computed tomography (CT) in the majority of cases (19, 20, 37), suggesting that these data may be overestimated. Moreover, there is no published data on the incidence of fatty and atheroma emboli. In our series, the incidence of SE is 3.6%, which is lower than that reported in our group’s previous study (6.2%) (33); we hypothesize that this may be due to the increased number of thrombi available from improved recanalization techniques over the years. Conversely, the incidence of CE is 0.9%, lower than that described in other series (20, 21), the incidence of AE is 1.1%, and the incidence of FE is 0.7% (with no incidence data available). Therefore, the incidence of atypical clot composition (septic, calcific, atheroma, and fatty emboli) in our sample is 6.25%.

Rapid recanalization is an essential aspect of the functional recovery for stroke patients treated with mechanical thrombectomy. Consequently, the composition and characteristics of the clot are crucial in guiding recanalization strategies and the development of endovascular devices. Factors associated with refractory thrombectomy include severe anatomical tortuosity, underlying pathologies such as intracranial stenosis or dissections, and atypical clot compositions, including SE or CE (36). In terms of procedural factors, we observed a higher number of passes during MT in the atypical clot group; however, no significant differences were noted in other procedural parameters, such as the first-pass effect and procedure duration.

In terms of vascular risk factors, DM and active smoking were found to be more frequently associated with atypical composition cases. The likely greater complexity of the procedure in the ACC group aligns with identifying DM as an independent predictor of refractory thrombectomy (36). The increased prevalence of DM may be linked to underlying vessel wall pathology (36), which, along with the unfavorable microstructural characteristics of atypical thrombi, contributes to the refractoriness of EVT and a poor long-term prognosis. Another independent factor related to the ACC group, probably associated with the increased complexity of EVT, is the higher incidence of occlusions in the intracranial carotid artery compared to UCC cases, indicative of a higher embolic load in ACC cases.

The number of leukocytes and 3-month mortality was higher in the ACC group of patients in the bivariate analysis, but no statistically significant difference was found in the multivariate analysis. As is widely known, infectious pathology of any origin is a risk factor for cerebral ischemia by facilitating the processes of thrombogenesis and atherosclerosis (39). Therefore, greater thrombotic material is expected in cases of SE, as we have already observed in our previous study (33).

Concerning functional parameters, although endocarditis is associated with high mortality rates (18) and the limited data available to date link CE to higher mortality compared with non-calcified thrombi (22), we found no significant differences between groups in the multivariate analysis. In the SE patients, there were no differences among any of the studied variables and the source of the infection. It is important to note that in most cases, no infectious focus was identified; therefore, we consider the low number of cases with known-origin infections a limitation, which could explain the lack of differences in our sample.

Finally, as described in the literature (40), the findings regarding neutrophil extracellular trap formation in all thrombi analyzed suggest that NETs are a common inflammatory component of thrombi in acute stroke.

Some limitations should be considered in this study. Given the lack of quantitative immunohistochemical analysis, we cannot make any conclusions regarding NET expression levels in atypical versus usual thrombi. Future studies focusing on quantitative immunohistochemical analysis would be important for addressing this question. Among other limitations, 25.7% of the patients were excluded due to an insufficient amount of clot material retrieved. This group likely includes patients with intracranial stenosis caused by calcified atheromatous plaques that adhere to the vascular wall, as well as those who underwent refractory thrombectomies, among others. The literature firmly establishes that fibrin-rich thrombi have lower recanalization rates; therefore, fibrin-rich thrombi may be underrepresented in our series. Finally, we emphasize that this is a retrospective study, which may introduce potential biases.

We highlight the detailed anatomopathological study conducted on a large cohort of patients as a notable strength in comparison to previously published studies.

## Conclusion

The strongest predictors of atypical clot composition in our series were DM and TICA occlusion. In addition, atypical clot composition is associated with a greater number of passes in MT. These findings suggest that the EVT procedure is likely more complex in cases involving atypical clot composition.

We were able to successfully identify factors associated with thrombus removal despite the lack of a specific classification for thrombotic material, which was attributed to the findings in our series and the proposed subdivision for our study. Therefore, we believe that our research may yield valuable insights for the management of such cases.

Our study’s NET findings suggest that the inflammatory component plays a vital role in the thrombotic process in ischemic stroke, which may also influence the outcome of the EVT procedure and potentially affect the future design of thrombus extraction devices.

## Data Availability

The raw data supporting the conclusions of this article will be made available by the authors without undue reservation.

## Glossary

AIS: Acute ischaemic stroke
ACA: Anterior cerebral artery
AE: Atheroma emboli
ACC: Atypical clot composition
BAS: Basilar
CE: Calcic emboli
Cit-H3: Citrullinated histone H3
CT: Cranial tomography
EVT: Endovascular treatment
FE: Fatty emboli
FPC: Fibrin/platelet-rich clots
HMCAS: Hyperdense middle cerebral artery sign
IE: Infective endocarditis
LVO: Large vessel occlusion
MT: Mechanical thrombectomy
MCA: Middle cerebral artery
MC: Mixed clots
MPO: Myeloperoxidase
NETs: Neutrophil extracellular traps
PCA-P1: Posterior cerebral artery P1 segment
PCA-P2: Posterior cerebral artery P2 segment
RBC: Red blood cells-rich clots
RT: Refractory thrombectomy
SE: Septic emboli
sICH: Symptomatic intracranial hemorrhage
TICA: Terminal internal carotid artery
UCC: Usual clot composition
WBC: White blood cells
